# Differential Regulation of GPVI-Induced Btk and Syk Activation by PKC, PKA and PP2A in Human Platelets

**DOI:** 10.3390/ijms24097776

**Published:** 2023-04-24

**Authors:** Pengyu Zhang, Fiorella A. Solari, Johan W. M. Heemskerk, Marijke J. E. Kuijpers, Albert Sickmann, Ulrich Walter, Kerstin Jurk

**Affiliations:** 1 Leibniz Institut für Analytische Wissenschaften-ISAS-e.V., 44139 Dortmund, Germany; 2 Center for Thrombosis and Hemostasis (CTH), University Medical Center of the Johannes Gutenberg University Mainz, 55131 Mainz, Germany; 3 Department of Biochemistry, CARIM, Maastricht University, 6229 ER Maastricht, The Netherlands; 4 Synapse Research Institute Maastricht, 6217 KD Maastricht, The Netherlands; 5 Medizinische Fakultät, Medizinisches Proteom-Center, Ruhr-Universität Bochum, 44780 Bochum, Germany; 6 Department of Chemistry, College of Physical Sciences, University of Aberdeen, Aberdeen AB25 2ZD, UK

**Keywords:** thrombo-inflammation, platelets, protein kinases, Bruton’s tyrosine kinase, XLA

## Abstract

Bruton’s tyrosine kinase (Btk) and spleen tyrosine kinase (Syk) are major signaling proteins in human platelets that are implicated in atherothrombosis and thrombo-inflammation, but the mechanisms controlling their activities are not well understood. Previously, we showed that Syk becomes phosphorylated at S297 in glycoprotein VI (GPVI)-stimulated human platelets, which limits Syk activation. Here, we tested the hypothesis that protein kinases C (PKC) and A (PKA) and protein phosphatase 2A (PP2A) jointly regulate GPVI-induced Btk activation in platelets. The GPVI agonist convulxin caused rapid, transient Btk phosphorylation at S180 (pS180↑), Y223 and Y551, while direct PKC activation strongly increased Btk pS180 and pY551. This increase in Btk pY551 was also Src family kinase (SFK)-dependent, but surprisingly Syk-independent, pointing to an alternative mechanism of Btk phosphorylation and activation. PKC inhibition abolished convulxin-stimulated Btk pS180 and Syk pS297, but markedly increased the tyrosine phosphorylation of Syk, Btk and effector phospholipase Cγ2 (PLCγ2). PKA activation increased convulxin-induced Btk activation at Y551 but strongly suppressed Btk pS180 and Syk pS297. PP2A inhibition by okadaic acid only increased Syk pS297. Both platelet aggregation and PLCγ2 phosphorylation with convulxin stimulation were Btk-dependent, as shown by the selective Btk inhibitor acalabrutinib. Together, these results revealed in GPVI-stimulated platelets a transient Syk, Btk and PLCγ2 phosphorylation at multiple sites, which are differentially regulated by PKC, PKA or PP2A. Our work thereby demonstrated the GPVI–Syk–Btk signalosome as a tightly controlled protein kinase network, in agreement with its role in atherothrombosis.

## 1. Introduction

Protein tyrosine kinases, in particular Src family kinases (SFKs), spleen tyrosine kinase (Syk) and Bruton’s tyrosine kinase (Btk), play central roles in the physiology and pathophysiology of immune and hematopoietic cells, including platelets [1,2]. In such cells, members of the SFK, Syk and Btk families become activated in response to stimulation of the membrane receptors, such as the B cell antigen receptor (BCR) [3], the T cell receptor (TCR) [4] and Fc receptors [5]. Receptor occupation commonly initiates intracellular signaling, leading to phosphorylation and activation of phospholipase Cγ (PLCγ) isoforms and activation of the Ca^2+^- and diacylglycerol (DAG)-dependent protein kinase C (PKC), followed by cell-specific responses, such as altered gene expression, bio-mediator release, integrin activation and cell proliferation. The tyrosine kinases Src, Syk and Btk have similar domain structures, although they represent distinct types of molecular switches [6].

Under basal conditions, Src, Syk and Btk are thought to be in a folded and auto-inhibited state, while their activation leads to an unfolding structure linked to tyrosine phosphorylation within the activation loops [6]. SFKs have an N-terminal myristoyl group that is responsible for the plasma membrane localization and is also required for the kinase activity. Upon membrane receptor stimulation, SFKs become activated due to a transphosphorylation of Y419 and dephosphorylation of the inhibitory site at Y530. SFKs are also phosphorylated by PKA, PKC and other kinases, which is a process that is less well-defined [6,7].

In unstimulated cells, Syk and Btk are also present in an autoinhibited state and require recruitment from the cytosol to the membrane for activation. With GPVI-mediated platelet activation, SFKs dually phosphorylate a receptor-associated ITAM (immunoreceptor tyrosine-based activation motif), which then recruits target proteins containing two SH2 domains, such as Syk. This unfolds the Syk kinase with simultaneous phosphorylation of its crucial tyrosine residues (Y348/352 and Y525/526), resulting in the activation of the kinase [2,8]. The activated Syk evokes multisite phosphorylation of the membrane-located protein linker adaptor for T-cells (LAT), which then recruits additional proteins, such as phospholipase Cγ2 (PLCγ2) and phosphoinositide 3-kinases (PI3K), via its SH2 domains [4]. The LAT-recruited PI3K leads to increased membrane levels of phosphatidylinositol (3,4,5)-trisphosphate (PIP_3_) with the recruitment of the PH-domain proteins PLCγ2, Btk, Akt and PDK1 [9]. Btk, membrane recruitment and subsequent phosphorylation of the Y551 residue in the activation loop are known to be required for its activation [1,10].

The kinase Btk was identified as the gene responsible for the human immunodeficiency disorder X-linked agammaglobinemia (XLA) [11,12]. The identified gene is expressed primarily in a variety of hematopoietic cells, including B cells, mast cells, macrophages and platelets. Most of the current understanding of Btk came from analysis of the B cell receptor signaling pathway [13]. In B cells, the signaling via SFKs, Syk and Btk affects a diverse set of downstream effector proteins, including PLCγ, PKC, PKA, Akt and mitogen-activated protein kinases (MAPKs), as well as several transcription factors [13,14]. Due to the increasingly recognized roles of BCR, Syk and Btk in diseases such as leukemia, lymphoproliferative and inflammatory/autoimmune disorders, potent inhibitors of Syk and Btk were developed over the last 15 years and several of them were validated and approved in clinical studies [3,14,15].

The kinases SFKs, Syk and Btk are central for human platelet activation, in particular in response to collagen, convulxin (Cvx) and other GPVI agonists. In early studies, Btk was shown to mediate collagen-induced PLCγ2 phosphorylation, Ca^2+^ mobilization from intracellular stores, dense granule secretion and aggregation of platelets, which were impaired in Btk-deficient platelets from XLA patients [16]. In a Btk-deficient murine model (Btk^xid^ animals), platelet functions in response to GPIb-V-IX (GPIb) were reduced [17]. Recent attention shifted to platelet Btk as a potential therapeutic target in platelet-related thrombotic diseases [18,19,20,21,22,23], but clinically used Btk inhibitors affecting platelet functions may also cause bleeding events [22,24]. While the principal mechanism of Btk activation is well-defined in platelets (membrane recruitment combined with tyrosine phosphorylation of the critical sites Y223 and Y551) [1,25], there are still important open questions concerning Btk activation and steady-state regulation by other protein kinases. Several post-translational modifications (PTMs) at serine (S), threonine (T) and tyrosine (Y) in terms of phosphorylation, acetylation and ubiquitylation were reported for both Syk and Btk (see the PhosphoSitePlus database) [1,2,26].

Previously, we demonstrated using a phosphoproteomic approach that the phosphorylation of Syk and Btk at S/T residues is regulated by both the platelet agonist adenosine diphosphate (ADP) and the platelet inhibitor prostaglandin I_2_ (PGI_2_) [27]. Subsequently, we demonstrated that the ADP- and GPVI-mediated activation of platelets results in PKC-dependent phosphorylation of a prominent S297 site in the Syk interdomain-B, which controls Syk activation [28,29]. Specifically, PKC inhibition reduces the Syk S297 phosphorylation, which leads to an enhanced tyrosine phosphorylation and activity of the kinase [28]. On the other hand, we found that the inhibition of protein phosphatase 2A (PP2A) increased Syk S297 phosphorylation, which led to reduced Syk tyrosine phosphorylation and activity [29]. These data support the concept that the Syk S297 phosphorylation/dephosphorylation by the key protein kinase (PKC) and phosphatase (PP2A) is a mechanism of regulatory cross-talk linked to tyrosine phosphorylation, and that it represents a feedback mechanism for controlling Syk activation in human platelets [30].

So far, similar studies concerning the tyrosine kinase Btk in platelets do not exist. With B cell lines, it was shown that the PKC-mediated phosphorylation of Btk S180 (in the N-terminal TH domain) negatively affects the Btk membrane localization and activity [31]. Here, we compared Syk and Btk activation in human platelets and showed that three Btk phospho-sites, namely, S180, Y223 and Y551, were regulated by GPVI and were additionally affected by PKC, as well as PKA signaling. GPVI-induced Btk Y551 phosphorylation and activation was transient and controlled PLCγ2 and platelet aggregation.

## 2. Results

### 2.1. GPVI Activation of Human Platelets Using Convulxin Transiently Increased Btk S180 and Y551 Phosphorylation with Slight Effects on Btk Y223

**Figure 1:**
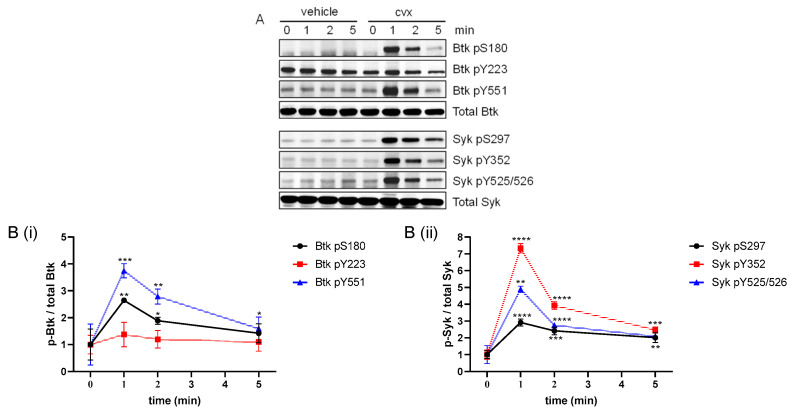
Cvx stimulation of platelets transiently upregulated Btk pS180, pY223 and pY551, along with Syk pS297, pY352 and pY525/526. Washed human platelets were treated with a vehicle (0.9% NaCl) or stimulated using 50 ng/mL Cvx with stirring, and stopped after 0, 1, 2 or 5 min with Lämmli buffer. (**A**) Representative blots of vehicle-treated or Cvx-stimulated Btk pS180, Btk pY223, Btk pY551, Syk pS297, Syk pY352 and Syk pY525/526. Antibodies against total Btk and Syk were used as loading controls. (**B**) The phosphorylation of Btk (**Bi**) and Syk (**Bii**) was analyzed and compared with total Btk and total Syk, respectively. Quantitative data are represented as the mean ± SD from three independent experiments with platelets from three healthy donors (n = 3). **** *p* < 0.0001, *** *p* < 0.001, ** *p* < 0.01, * *p* < 0.05, 0 min versus 1 min, 2 min and 5 min in response to Cvx-treated platelets.

We previously described and characterized the fact that GPVI induces phosphorylation of Syk serine 297 (Syk pS297), tyrosine 352 (pY352) and tyrosine 525/526 (pY525/526) in studies with Cvx-stimulated human platelets [28]. In this study, we characterized Cvx (50 ng/mL)-regulated serine and tyrosine phosphorylation of Btk (S180, Y223 and Y551) and compared this directly to the serine and tyrosine phosphorylation of Syk using different and specific phospho-site antibodies. In agreement with our previous data, Cvx induced a rapid, strong and essentially irreversible platelet aggregation (Supplementary Materials Figure S1a). Except for only slight effects on Btk Y223, all Btk and Syk phospho-sites increased using Cvx were rapid and transient (Figure 1). Btk Y551 phosphorylation (Btk pY551) is required for Btk activation and was widely used as an indicator of Btk activation in immune cells and platelets, whereas the role of the autophosphorylation site (Btk Y223) is less clear. Here we show for the first time that Cvx rapidly induced Btk S180 phosphorylation (Btk pS180), which paralleled with Btk activation (Btk pY551) and resembled the pattern of Syk phosphorylation.

### 2.2. Differential Regulation of Platelet Btk and Syk Phosphorylation by the PKC Activator PDBu

**Figure 2:**
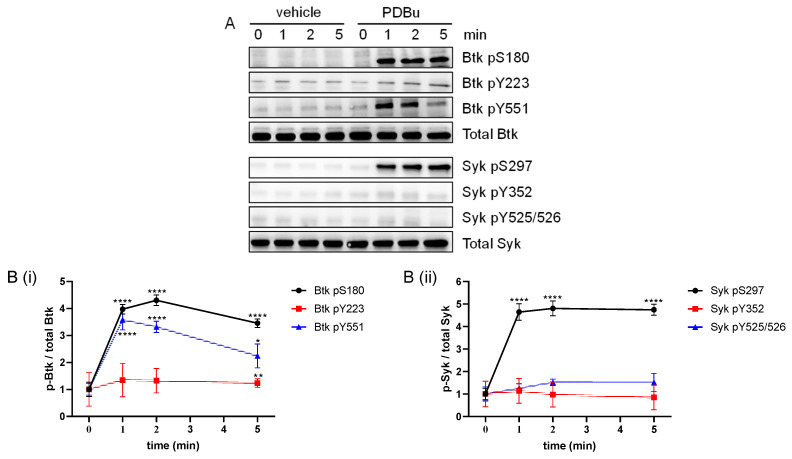
The PKC activator PDBu upregulated the platelets Btk pS180, Btk pY551 and Syk pS297. Washed human platelets were treated with a vehicle (0.1% DMSO) or stimulated using 0.2 µM PDBu with stirring and stopped after 0, 1, 2 or 5 min using Lämmli buffer. (**A**) Representative blots of the vehicle/PDBu-treated platelets showing Btk pS180, Btk pY223, Btk pY551, Syk pS297, Syk pY352 and Syk pY525/526. Antibodies against total Btk and Syk were used as loading controls. (**B**) The phosphorylation of Btk (**Bi**) and Syk (**Bii**) was analyzed and compared with the total Btk and total Syk, respectively. Quantitative data are represented as the mean ± SD from three independent experiments with platelets from three healthy donors (n = 3). **** *p* < 0.0001, ** *p* < 0.01, * *p* < 0.05, 0 min versus 1 min, 2 min and 5 min in response to PDBu-treated platelets.

Previously, enhanced Syk pS297 in platelets was also observed with the protein kinase C (PKC) activator phorbol 12,13-dibutyrate (PDBu) [28]. To test the hypothesis that PKC may also regulate Btk pS180, washed platelets were incubated with 0.2 µM PDBu in comparison to a vehicle control (0.1% DMSO). As expected, PDBu induced a strong aggregation of platelets (Supplementary Materials Figure S1b), which was accompanied by a consistent Syk pS297 (Figure 2A,(Bii)). In addition, PDBu strongly and persistently induced the Btk pS180. Surprisingly, it also transiently upregulated Btk pY551 and slightly upregulated Btk Y223 phosphorylation (Btk pY223) (Figure 2A,(Bi)), but not Syk tyrosine phosphorylation (Figure 2A,(Bii)). These new data demonstrate a differential regulation of Btk and Syk by PDBu, and led us to examine the mechanism of PDBu-induced Btk Y551 phosphorylation.

### 2.3. PDBu-Induced Phosphorylation of Btk at Y551 Was Antagonized by the PKC Inhibitor GF109203X and SFK Inhibitor PP2 but Not by the Inhibition of Syk or Btk

**Figure 3:**
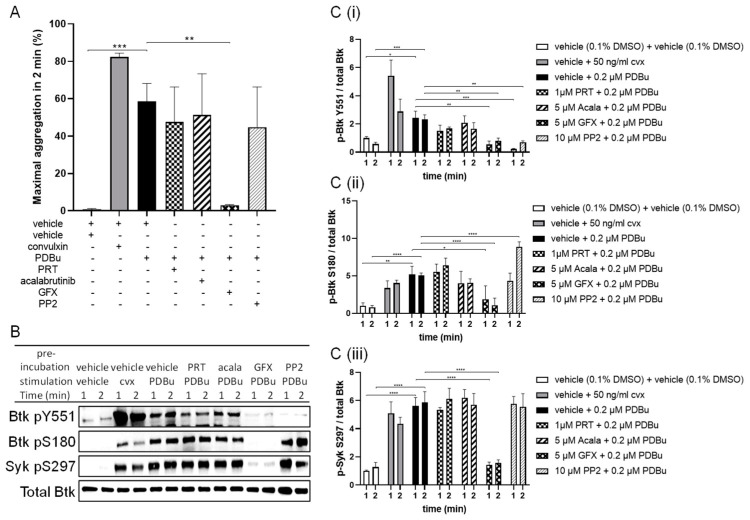
The PDBu-induced phosphorylation of Btk Y551 was abolished by GFX and PP2. Washed human platelets were treated with a vehicle (0.1% DMSO) or 1 µM PRT-060318 (PRT), 5 µM acalabrutinib, 5 µM GFX or 10 µM PP2 prior to stimulation with 50 ng/mL Cvx or 0.2 µM PDBu with stirring. Basal platelet samples without PDBu or Cvx stimulation were incubated with the appropriate concentration of 0.1% or 0.2% DMSO vehicle. Platelet aggregation was stopped after 1 and 2 min by Lämmli buffer. (**A**) Maximal aggregation values at 2 min of human platelets stimulated by Cvx or PDBu. (**B**) Representative blots showing the effects of Cvx, PDBu and PDBu/inhibitor combinations on Btk pY551, Btk pS180 and Syk pS297, with total Btk as the loading control. (**C**) The phosphorylation of Btk Y551 (**Ci**), Btk S180 (**Cii**) and Syk S297 (**Ciii**) was analyzed and compared with total Btk. Quantitative data are represented as the mean ± SD from three independent experiments with platelets from three healthy donors. **** *p* < 0.0001, *** *p* < 0.001, ** *p* < 0.01, * *p* < 0.05, vehicle versus PRT/acalabrutinib/GFX/PP2-treated platelets in response to PDBu at the same time.

There is evidence of Src-PKC interactions and that PKC may induce Src activation/phosphorylation [7]. To further identify the cause of PDBu-induced Btk phosphorylation, we examined the effect of the inhibitors of SFK, Syk, Btk and PKC on PDBu-induced Btk pY551, Btk pS180 and Syk pS297. Washed human platelets were then stimulated with PDBu or Cvx after preincubation with either a vehicle or SFK inhibitor PP2, Syk inhibitor PRT-060318 (PRT), Btk inhibitor acalabrutinib or PKC inhibitor GF109203X (GFX) for 5 min. GFX, but not PRT/acalabrutinib/PP2, inhibited the PDBu-induced platelet aggregation (Figure 3A). Both GFX and PP2 blocked the PDBu-induced Btk phosphorylation at Y551 (Figure 3B,(Ci)). Markedly, of these inhibitors, only GFX blocked the PDBu-induced Btk pS180 and Syk pS297 (Figure 3B,(Ciii)).

### 2.4. Activation of PKC Enhanced and Prolonged the Convulxin-Induced Btk S180 Phosphorylation with a Minor Effect on Tyrosine Phosphorylation

**Figure 4:**
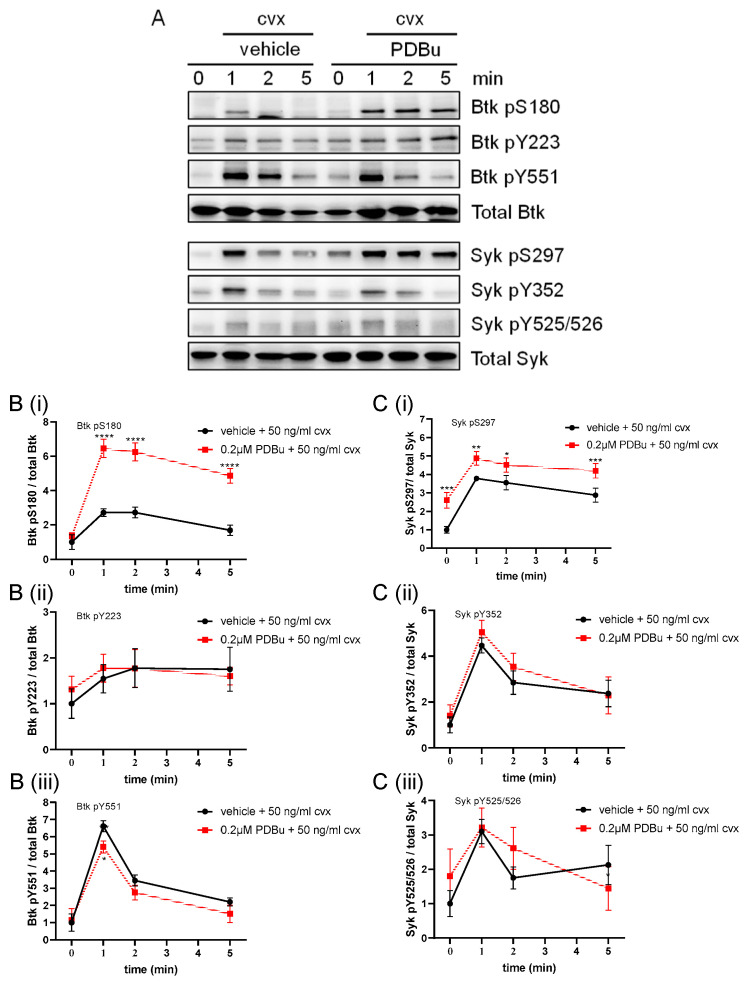
The PKC activator PDBu upregulated Cvx-induced phosphorylation of Btk S180 and Syk S297. Washed human platelets were pre-incubated with a vehicle (0.1% DMSO) or 0.2 µM PDBu for 1 min; then stimulated using 50 ng/mL Cvx with stirring; and stopped after 0, 1, 2 or 5 min using Lämmli buffer. (**A**) Representative blots of Btk pS180, Btk pY223, Btk pY551, Syk pS297, Syk pY352 and Syk pY525/526. Antibodies against total Btk and Syk were used as loading controls. (**B**) Phosphorylation of Btk at S180 (**Bi**), Y223 (**Bii**) and Y551 (**Biii**) was analyzed and compared with total Btk. (**C**) Phosphorylation of Syk at S297(**Ci**), Y352 (**Cii**) and Y525/526 (**Ciii**) was analyzed and compared with total Syk. Quantitative data are represented as the mean ± SD from three independent experiments with platelets from three healthy donors (n = 3). **** *p* < 0.0001, *** *p* < 0.001, ** *p* < 0.01, * *p* < 0.05, DMSO versus PDBu-treated platelets in response to Cvx at the same time.

It was interesting to examine the interaction of PDBu- and Cvx-regulated Btk pS180. We analyzed the effects of PDBu on Cvx-regulated Btk and Syk phosphorylation. Platelets pre-incubated with the vehicle control (0.1% DMSO) or with 0.2 PDBu for 1 min prior to Cvx (50 ng/mL) stimulation induced a similar aggregation response compared with aggregation using Cvx alone (50 ng/mL) (Supplementary Materials Figure S1c). However, PDBu strongly increased and prolonged the Cvx-induced Btk pS180 and Syk pS297, whereas the tyrosine phosphorylation did not differ (Figure 4). These data suggest that PDBu/PKC increased the Cvx-induced Syk pS297 and Btk pS180. We then tested the hypothesis that the Cvx-induced Btk pS180 was mediated by PKC.

### 2.5. PKC Inhibition Abolished the Convulxin-Induced Btk S180 and Syk S297 Phosphorylation but Enhanced the Btk Y551 and Syk Y525/526 Phosphorylation

**Figure 5:**
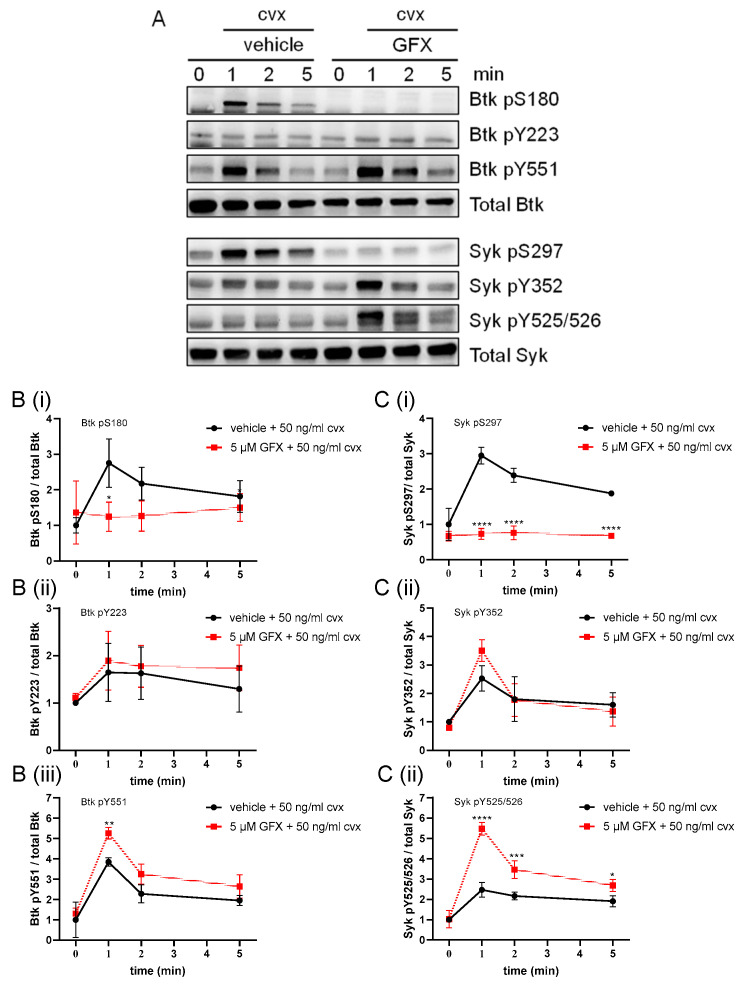
PKC inhibition strongly reduced the Cvx-induced phosphorylation of Btk S180 and Syk S297 but enhanced both Btk Y551 and Syk Y525/526 phosphorylation. Washed human platelets were treated with a vehicle (0.1% DMSO) or 5 µM GF109203X (GFX) for 5 min prior to stimulation with 50 ng/mL Cvx with stirring. Platelet aggregation was stopped after 0, 1, 2 or 5 min using Lämmli buffer. (**A**) Representative blots of Btk pS180, Btk pY223, Btk pY551, Syk pS297, Syk pY352 and Syk pY525/526. Antibodies against total Btk and Syk were used as loading controls. (**B**) The phosphorylation of Btk S180 (**Bi**), Y223 (**Bii**) and Y551 (**Biii**) was analyzed and compared with total Btk. (**C**) The phosphorylation of Syk S297 (**Ci**), Y352 (**Cii**) and Y525/526 (**Ciii**) was analyzed and compared with total Syk. Quantitative data are represented as the mean ± SD from three independent experiments with platelets from three healthy donors (n = 3). **** *p* < 0.0001, *** *p* < 0.001, ** *p* < 0.01, * *p* < 0.05, DMSO versus GFX-treated platelets in response to Cvx.

Previously, we observed that GPVI-evoked Syk pS297 was blocked by the established PKC inhibitor GF109203X (GFX) [28]. Therefore, the effect of GFX on Cvx-indued Btk phosphorylation was analyzed in comparison to Syk phosphorylation. In line with previous results, the GFX treatment partially inhibited the Cvx-induced aggregation response (Supplementary Materials Figure S1d). GFX completely abolished the Cvx-induced Btk pS180 and Syk pS297, but enhanced the Cvx-induced Btk pY551, Syk pY525/526 and Syk pY352 (slightly) (Figure 5). These data provide evidence that Btk pS180 in Cvx-stimulated platelets was sensitive to PDBu and GFX and hence was mediated by PKC. Knowing that the cAMP-dependent stimulation of PKA strongly inhibits PKC activation in human platelets [28], we investigated a possible role of PKA in Btk regulation.

### 2.6. PKA Activation Abolished the Convulxin-Induced Btk S180 and Syk S297 Phosphorylation and Moderately Enhanced the Tyrosine Phosphorylation Events

**Figure 6:**
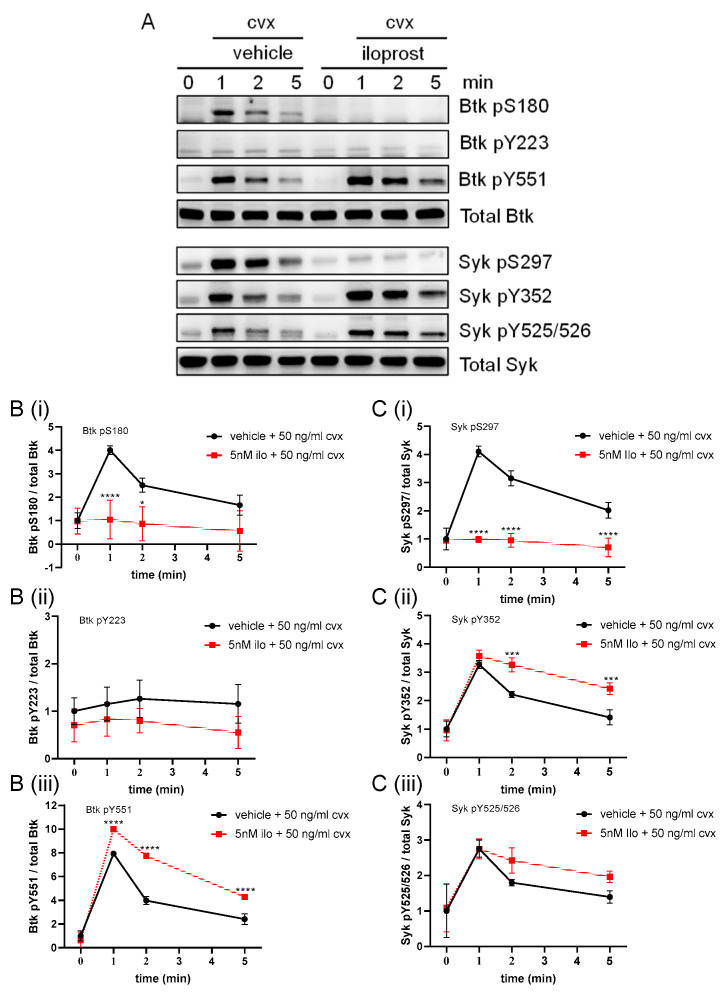
PKA activation inhibited the Cvx-induced phosphorylation of Btk S180 and Syk S297 but enhanced the tyrosine phosphorylation of Btk and Syk. Washed human platelets were treated with a vehicle (0.9% NaCl) or 5 nM iloprost for 5 min prior to stimulation with 50 ng/mL Cvx with stirring. Platelet aggregation was stopped after 0, 1, 2 or 5 min using Lämmli buffer. (**A**) Representative blots of Btk pS180, Btk pY223, Btk pY551, Syk pS297, Syk pY352 and Syk pY525/526. Antibodies against total Btk and Syk were used as loading controls. (**B**) The phosphorylation of Btk at S180 (**Bi**), Y223 (**Bii**) and Y551 (**Biii**) was analyzed and compared with total Btk. (**C**) The phosphorylation of Syk at S297 (**Ci**), Y352 (**Cii**) and Y525/526 (**Ciii**) was analyzed and compared with total Syk. Quantitative data are represented as the mean ± SD from three independent experiments with platelets from three healthy donors (n = 3). **** *p* < 0.0001, *** *p* < 0.001, * *p* < 0.05, DMSO versus iloprost-treated platelets in response to Cvx.

We showed earlier that activation of PKA by the prostacyclin analog iloprost abolished the ADP- and Cvx-induced platelet aggregation responses, as well as PKC activation, whereas iloprost did not inhibit the Cvx-induced activatory Syk tyrosine phosphorylation [27,28]. Here, we investigated the PKA effects on Cvx-induced Btk phosphorylation changes. As required, iloprost blocked the Cvx-induced platelet aggregation (Supplementary Materials Figure S1e). Iloprost acting via PKA prevented the Cvx-induced Btk pS180 and Syk pS297, but it increased Btk pY551, Syk pY352 and pY525/526 (slightly) (Figure 6). This indicated that the effects of PKA activation on Syk S297 and Btk S180/Y551 phosphorylation were similar to those of PKC inhibition.

### 2.7. Inhibition of PP2A Increased the Syk S297 but Not the Btk S180 Phosphorylation

**Figure 7:**
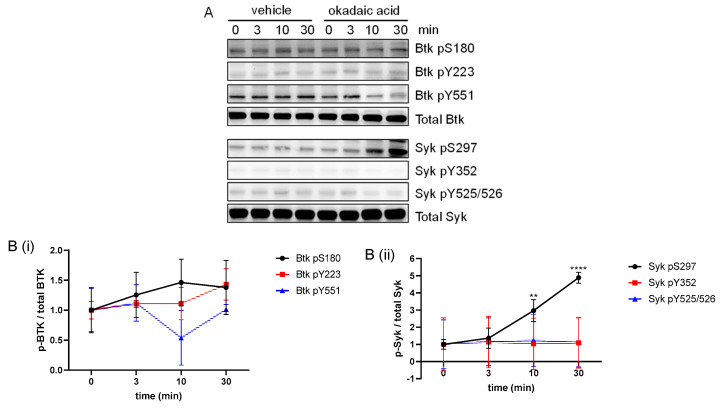
The PP2A inhibitor okadaic acid (OA) upregulated platelet Syk S297 phosphorylation but not the phosphorylation of Btk S180 or any other Syk/Btk phospho-site. Washed human platelets were treated with a vehicle (0.1% ethanol) or with 1 µM OA under stirring and stopped after 0, 3, 10 or 30 min using Lämmli buffer. (**A**) Representative blots of vehicle- or OA-treated Btk pS180, Btk pY223, Btk pY551, Syk pS297, Syk pY352 and Syk pY525/526. Antibodies against total Btk and Syk were used as loading controls. (**B**) The phosphorylation of Btk (**Bi**) and Syk (**Bii**) was analyzed and compared with total Btk and total Syk, respectively. Quantitative data are represented as the mean ± SD from three independent experiments with platelets from three healthy donors (n = 3). **** *p* < 0.0001, ** *p* < 0.01, 0 min versus 3 min, 10 min and 30 min in response to OA-treated platelets.

Previously, we reported that inhibition of the serine/threonine protein phosphatase 2A (PP2A) using okadaic acid (OA) induced a stoichiometric Syk pS297. In addition, OA was found to inhibit the Cvx-induced tyrosine phosphorylation of Syk, LAT and PLCγ2 [29]. First, we confirmed that PP2A inhibition alone strongly elevated the Syk S297 phosphorylation. Interestingly, this treatment did not induce platelet aggregation (Supplementary Materials Figure S1f). In contrast, PP2A inhibition alone did not affect the Btk pS180 or Syk/Btk tyrosine phosphorylation events (Figure 7). These results indicated a direct role of PP2A in the regulation of Syk pS297 but not of Btk pS180 in resting platelets.

### 2.8. Btk Inhibition Abolished While PKC Inhibition Enhanced the Cvx-Induced PLCγ2 Phosphorylation

**Figure 8:**
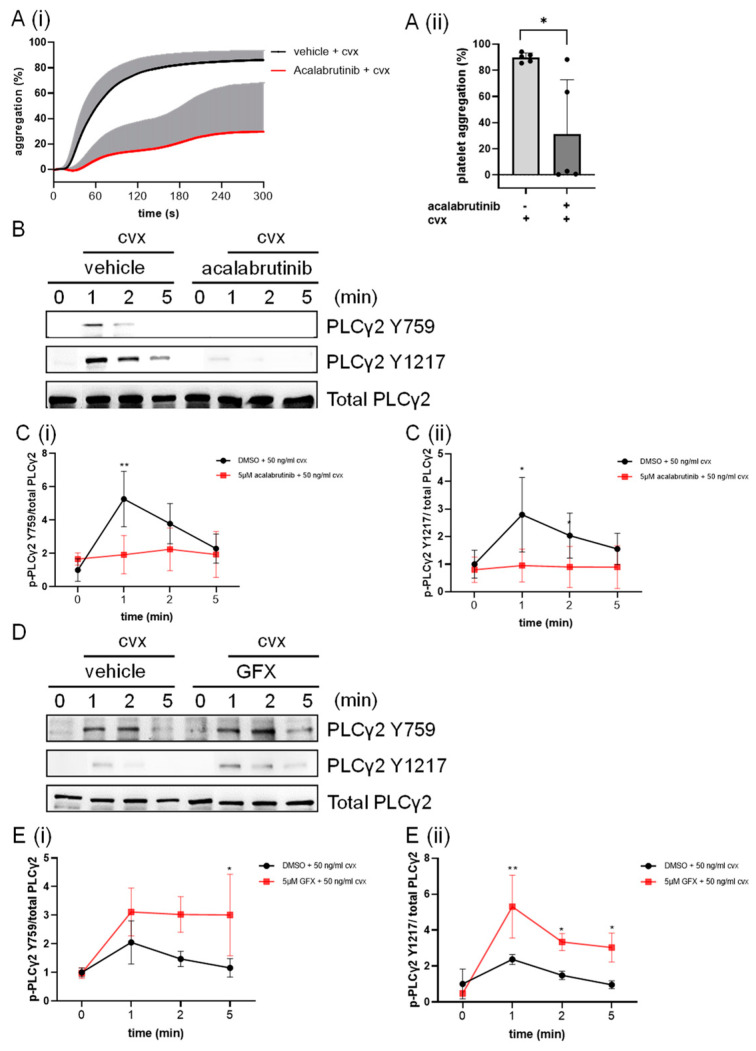
Phosphorylation of PLCγ2 at Y759 and Y1217 induced using Cvx was abolished by the Btk inhibitor acalabrutinib and enhanced, as well as prolonged by the PKC inhibitor GF109203X (GFX, 5 µM). Washed human platelets were treated with a vehicle (0.1 % DMSO) or 5 µM acalabrutinib or 5 µM GFX for 5 min prior to stimulation with 50 ng/mL Cvx with stirring. Platelet aggregation was stopped after 0, 1, 2 or 5 min using Lämmli buffer. (**A**) Time dependent platelet aggregation (**Ai**) and maximal platelet aggregation (**Aii**) within 5 min induced by 50 ng/ml Cvx after pre-incubation with vehicle (0.1% DMSO) or 5 µM acalabrutinib.Data are represented as the mean + SD from five healthy donors. (**B**) Representative blots of PLCγ2 Y759, Y1217 and total PLCγ2 (loading control) stimulated by Cvx after pre-incubation with acalabrutinib. (**C**) The phosphorylation of PLCγ2 Y759 (**Ci**) and Y1217 (**Cii**) was analyzed and compared with total PLCγ2. (**D**) Representative blots of PLCγ2 Y759, Y1217 and total PLCγ2 (loading control) stimulated by Cvx after pre-incubation with GFX. (**E**) The phosphorylation of PLCγ2 Y759 (**Ei**) and Y1217 (**Eii**) was analyzed and compared with total PLCγ2. Quantitative data are represented as the mean ± SD from three independent experiments with platelets from three healthy donors. ** *p* < 0.01, * *p* < 0.05, DMSO versus acalabrutinib-treated or GFX-treated platelets in response to Cvx.

Considering that PLCγ2 is a regulated substrate of Btk, we analyzed the effects of the Btk inhibitor acalabrutinib and PKC inhibitor GF109203X (GFX) on the Cvx-induced phosphorylation of PLCγ2. Out of five donors, acalabrutinib completely abolished the Cvx-induced platelet aggregation response for three donors, and partially inhibited the response for two donors (Figure 8A). As already mentioned in Section 2.5, GFX partially inhibited the Cvx-induced aggregation (Supplementary Materials Figure S1d). Furthermore, acalabrutinib abolished the Cvx-stimulated PLCγ2 Y759 and Y1217 phosphorylation (Figure 8B,C). In marked contrast, GFX strongly increased and prolonged the Cvx-induced PLCγ2 Y759 and Y1217 phosphorylation events (Figure 8D,E).

## 3. Discussion

**Figure 9:**
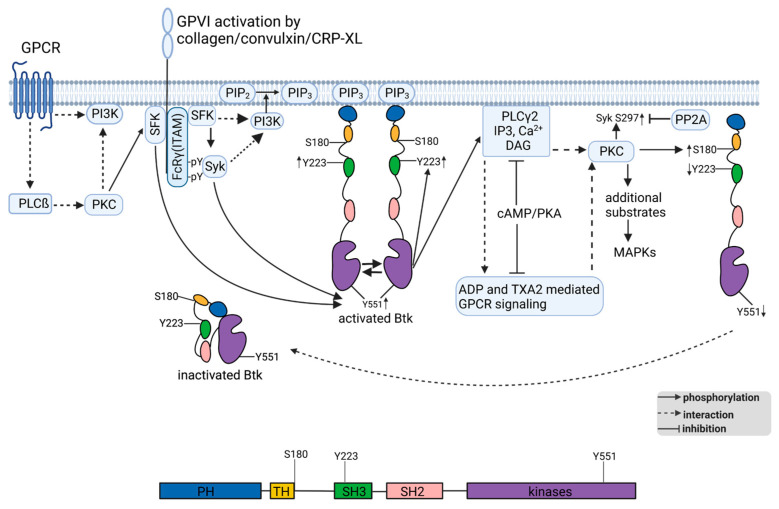
Model showing the domain organization, transient activation and regulation of Btk by SFKs, PI3K, PKC, PKA and PP2A. GPVI agonists activate platelets initiated by SFKs, which dually phosphorylate the immunoreceptor tyrosine-based activation motifs (ITAM) of the GPVI-coupled FcRγ chain and thereby recruit and activate Syk via its SH2 domains. GPVI-stimulated SFKs, Syk and GPCRs activate members of the PI3K family. This converts PIP_2_ to PIP_3_ and thereby recruits Btk from the cytosol to the membrane associated with its dimerization. Btk tyrosine phosphorylation of Y223 (autophosphorylation) and Y551 by SFKs and/or Syk leads to a fully activated kinase. Consequently, activation of the Btk substrate PLCγ2 results in the generation of IP_3_, Ca^2+^ and DAG, activation of PKC, and release of ADP and thromboxane A2 (TXA2). These secondary mediators enhance platelet activation via specific GPCRs, which also activate PLCβ and distinct PKC isoforms that induce the activation of SFKs. The Btk Y551 phosphorylation increased by GPCR stimulation/PKC/SFKs is independent of Syk and represents an alternative mechanism of Btk activation. GPVI activation not only induces Btk tyrosine phosphorylation but also Btk S180 phosphorylation, which is located in the N-terminal to the Btk TH domain. GPVI-induced Btk S180 phosphorylation is mediated by PKC and is associated with decreased Btk Y551 phosphorylation/activation. Elevation of cAMP levels and PKA activation reduce PKC responses. PP2A antagonizes Syk S297 phosphorylation but not Btk S180 phosphorylation. We propose that Btk S180 phosphorylation represents an important mechanism for Btk feedback inhibition in platelets. Figure was created using Biorender.com.

This paper reports several novel aspects of platelet collagen receptor GPVI-stimulated Btk activation, including tyrosine (Y223, Y551) and serine (S180) phosphorylation, which are rapid, reversible and regulated by PKC, PKA and PP2A. The SH3-domain-located Btk Y223, which was not strongly regulated here, is an established autophosphorylation site, which may recruit proteins containing the SH2 domain or regulate the ligand-binding property of the Btk [1]. Btk Y551, which is located in the activation loop, is essential for Btk protein kinase activity and BCR function in immune cells [6,32] and, when phosphorylated (pY511), reflects Btk activation. Btk itself (autophosphorylation), SFKs and Syk have the capacity to phosphorylate Btk Y551 [1,6,10]. In intact cells, Btk activation additionally requires its PH-domain mediated membrane recruitment to PI3K-generated PIP3 (Figure 9).

Here, we showed that GPVI stimulation transiently increased phosphorylation of both Btk S180 and Y551, which was strongly stimulated within 1 min and then rapidly reversed within 5 min, whereas Cvx-stimulated platelet aggregation was not reversed. Consistent with previous studies [28], the phosphorylation and activation of Syk (Y352, Y526/526 and S297) was also rapid and reversed. Protein levels of both Btk and Syk (shown as total Btk and Syk) were not affected, ruling out the idea that decreased tyrosine phosphorylation was caused by the degradation of Btk or Syk. Protein tyrosine phosphatases were therefore the main candidates responsible for the transient Y-phosphorylation. While there is substantial evidence that protein tyrosine phosphatases dephosphorylate components of GPVI signaling (SFKs, Syk) [33,34,35], very little is known about tyrosine phosphatases that use Btk pY551 as a direct substrate in platelets or other cells. However, tyrosine phosphatases that control SFK–Syk signaling (SHP1, SHP2 or TULA) may indirectly target Btk Y551, as reported for TULA-2 deficient murine platelets [36]. Importantly, transient tyrosine phosphorylation (Btk Y551, Syk Y352 and Y525/526) during GPVI stimulation is well reflected by the phosphorylation state of the Syk substrate LAT [28] and the Btk substrate PLCγ2 (Figure 8).

Another novel result of this study was that Btk S180, which is located within the N-terminal regulatory TH domain (see Figure 9), is a substrate for both Cvx- and PDBu-stimulated PKCs, similar to Syk S297. As reported previously, PKC inhibition abolished Cvx-induced Syk pS297 but enhanced Syk tyrosine phosphorylation/activity [28]. An earlier study demonstrated that inhibition of PKC enhanced GPVI-mediated Syk tyrosine phosphorylation (hyperphosphorylation) in human platelets, but not in murine platelets [37]. Here, we showed that the selective PKC inhibitor GF109203X (GFX) abolished Cvx-induced Btk pS180 but enhanced Cvx-induced Btk pY551. Earlier, Btk pS180 was discovered as a substrate for PKCß in murine and human B cell lines [31]. This study also showed that Btk S180 phosphorylation inhibited its membrane localization and downregulated BCR signaling, which was reversed by PKC inhibition. Furthermore, enhanced Btk Y-phosphorylation, membrane localization and BCR signaling was observed with a Btk S180A mutant in B cell lines. These data [31] and our present results suggest that PKC-mediated Btk pS180, due to its TH domain location, inhibited the PH-domain-dependent Btk recruitment to the cell membrane in Cvx-activated human platelets, resulting in decreased Btk Y551 phosphorylation/activation, which was partially reversed by PKC inhibition. Similarly, PKA stimulation, which strongly inhibits receptor-mediated PKC activation in human platelets [27,38], decreased PKC-mediated effects on both Syk S297 and Btk S180 phosphorylation.

A further novel finding was that PDBu alone significantly and also transiently increased the Btk pY551 without affecting any other Syk or Btk tyrosine phospho-sites. We then showed that PDBu-induced Btk pY551 was strongly inhibited by GFX and, unexpectedly, also by the Src/SFK inhibitor PP2. In contrast, Syk inhibitor PRT-060318 and Btk inhibitor acalabrutinib had only a little, if any, effect. PDBu-induced Syk pS297 and Btk pS180 were abolished only by GFX but not by PP2 or other tyrosine kinase inhibitors. Moreover, PDBu-stimulated aggregation of washed human platelets was abolished by GFX but not affected by Src/SFK, Syk and Btk inhibitors, indicating that Syk and Btk were not required for this PDBu/PKC aggregation response. PDBu-induced Syk pS297/Btk pS180 was directly PKC-mediated, whereas the PDBu-induced Btk pY551 was mediated by a PKC-stimulated Src/SFK without the participation of Syk. Interestingly, bidirectional phosphorylation/activation of PKCs and SFKs was well reported and described for platelets and other cell types [7]. Furthermore, a GPCR (thrombin, ADP) or phorbol 12-myristate 13-acetate (PMA)-activated PKC was shown to stimulate Src, which then phosphorylated RapGEF1 (C3G) at Tyr504, resulting in its activation and enhanced platelet aggregation [39].

Btk can also be activated by chemokine receptor-coupled G-proteins and their dissociated Gα and Gβγ subunits, which may bind directly to PI3K or even Btk, resulting in the activation of Btk and Akt [13]. We recently observed a chemokine-induced Btk pY551 with human platelets [40]. Our data suggest the presence of at least two distinct pathways that stimulated Btk Y551 phosphorylation and activation in human platelets. The first, established pathway was initiated by GPVI (GPIb, CLEC-2) and mediated by SFKs and Syk. The second, alternative pathway described now consisted of GPCRs (thrombin, ADP), PLCβ-mediated PKC activation and, subsequently, Src/SFKs. These two Btk-activating pathways may contain distinct SFK and PKC members, which requires future studies.

Cvx-stimulated phosphorylation of Syk S297 and Btk S180 was also transient and almost completely reversed after 5 min of stimulation, indicating the activity of potent serine/threonine protein phosphatases. Earlier, we established PP2A as a major Syk S297 protein phosphatase in platelets [29]. Here, PP2A inhibition by OA alone did not affect Btk pS180, suggesting the involvement of another serine/threonine protein phosphatase, which was abundantly expressed in human platelets [41]. We concluded that Btk was not directly affected by PP2A/OA. However, PP2A/OA affected Btk activity and its downstream substrate PLCγ2 indirectly via Syk since Cvx/OA combinations enhanced Syk pS297 but decreased Syk and Btk activity, as expressed by reduced LAT pY220 and PLCγ2 pY759, respectively [29].

The functional effects of Btk are defined primarily by its substrates, although Btk and other tyrosine kinases have also a role as adapter proteins [1]. A crucial substrate present in immune cells and platelets is PLCγ2 (or PLCγ1), which is phosphorylated by Btk on four distinct, highly conserved and important tyrosine sites (Y753, Y759, Y1197 and Y1217), resulting in increased enzymatic activity with DAG/IP_3_/Ca^2+^ elevation and PKC activation [42]. Here, the Cvx-induced PLCγ2 phosphorylation at Y759 and Y1217 was rapid, transient, enhanced and prolonged by the PKC inhibitor GFX, and abolished by the Btk inhibitor acalabrutinib, which also inhibited platelet aggregation as the final functional response (Figure 8). Earlier, we reported that several Btk inhibitors, including acalabrutinib, suppressed GPVI-induced aggregation and the secretion of human platelets [43]. We also showed that GFX enhanced GPVI-induced intracellular inositol monophosphate and Ca^2+^ mobilization in human platelets, indicating increased PLCγ2 activity [28]. We concluded for GPVI-activated human platelets that Btk was the major kinase responsible for PLCγ2 tyrosine phosphorylation/activation, which paralleled that of Btk tyrosine phosphorylation/activation. PLCγ2 activation then led to the activation of the PKC family, MAPKs, Ca^2+^/calmodulin and/or Ca^2+^-dependent pathways.

Btk is an important component of platelet activation mediated by GPVI, GPIb-IX-V, CLEC-2 and certain GPCRs, which often contribute to “hyperactive platelet disorders”, atherothrombosis and thrombo-inflammation [21]. However, activation of Syk, Btk and PLCγ2 in human platelets is not a simple linear, all-or-nothing process, but has multiple interactions and may reach levels of low, medium and high reactivity, which are controlled by many additional factors and signaling pathways (Figure 9). PKC and PKA up- and downregulate Btk S180 phosphorylation, respectively, and modulate the extent of Btk Y551 and PLCγ2 phosphorylation/activity. It is remarkable that PKA, which is one of the most potent inhibitory protein kinases in platelets, does not inhibit, but often enhances, Cvx-induced tyrosine phosphorylation of Syk, Btk, LAT and PLCγ2, whereas the more downstream events, such as PKC-mediated Syk pS297/Btk pS180 and Akt activation, are strongly inhibited [28,44]. There are still many unsolved questions, such as the precise mechanism of Btk membrane recruitment, identity of involved protein phosphatases, adapter proteins, and role of additional Btk phosphosites. Syk, Btk and PLCγ2 are essential components of the GPVI-induced platelet signalosome, which is implicated in atherothrombosis and thrombo-inflammation [21,23,45]. It will be important to unravel the fine-tuning of platelet SFK–Syk–Btk–PLCγ2 signaling and functional responses under physiological and pathophysiological conditions.

Finally, multiple genetic receptor variants, which signal via platelet SFKs, are known [46]. Recently, an important paper reported six patients with monoallelic missense gain-of-function Syk mutations within the kinase domain. These patients had recurrent infections, inflammation and a propensity to develop diffuse B cell lymphomas [47]. Furthermore, enhanced Btk protein expression and Y551 phosphorylation/activity was observed in patients with autoimmune diseases [48], which needs to be extended to platelet signaling. Hyperactive platelets, due to enhanced GPVI-Syk-Btk signaling, could be controlled in the future by novel inhibitors of protein kinases, such as Syk, PI3K, Btk, PKC and MAPKs [49,50,51], which are well expressed in human platelets

## 4. Materials and Methods

### 4.1. Materials

Platelet activators/inhibitors: Convulxin (Cvx), GF 109203X (GFX) and okadaic acid (OA) were obtained from Enzo Life Sciences (Lausen, Switzerland). Phorbol ester phorbol 12, 13-dibutyrate (PDBu) was supplied by Sigma-Aldrich (Saint Louis, MO, USA). Iloprost was obtained from Ratiopharm (Ulm, Germany). Acalabrutinib and PP2 were purchased from Abcam (Cambridge, UK). PRT-060318 was from Sellckem (Houston, TX, USA).

Sources of antibodies: General Syk antibody was from Sigma-Aldrich (Saint Louis, MO, USA). General Btk, Btk pS180, Btk pY223, Btk pY551, Syk pS297, Syk pY352, Syk pY525/526, general PLCγ2, PLCγ2 pY759 and PLCγ2 pY1217 antibodies were provided by Cell Signaling Technologies (Danvers, MA, USA). Secondary horseradish peroxidase (HRP)-conjugated goat anti-rabbit/mouse antibodies were obtained from BioRad Laboratories (Hercules, CA, USA).

Reagents: Tween^®^-20 and Precision Plus Protein Dual Color Standard was Bio-Rad Laboratories (Feldkirchen, Germany). Bovine serum albumin (BSA) was from Capricorn Scientific (Ebersdorfergrund, Germany). Clarity^TM^ Western ECL Substrate and β- mercaptoethanol were from BioRad Laboratories (Hercules, CA, USA). D(+)-Glucose, ethanol (EtOH), ethylene glycol-bis (β-aminoethyl ether)-N,N,N′,N′-tetraacetic acid (EGTA), glycerol, hepes and sodium chloride (NaCl) were from Carl Roth (Karlsruhe, Germany). Dimethyl sulfoxide (DMSO) was from Merck (Darmstadt, Germany). 

Equipment: Apact4S Plus aggregometer was from DiaSys Greiner (Flacht, Germany). Fusion FX7 apparatus was from Vilber Loumat (Eberhardzell, Germany). Automatic Haematology analyser Sysmex® KX-21N was from Sysmex Corporation Japan/Sysmex Germany. Centrifuge Allegra X-30R (rotors F2402, SX4400) was from Beckmann Coulter (Krefeld, Germany). 

### 4.2. Blood Donors, Ethics Approval and Informed Consent

Blood was collected from healthy volunteers without taking platelet-affecting drugs and without presenting inflammatory, infectious or allergic symptoms for at least 14 days, including normal blood counts. Informed consent was obtained from all healthy donors prior to participation in the study. This study was approved in accordance with the Declaration of Helsinki by the local Ethics Committee of the University Medical Center Mainz, as detailed below.

### 4.3. Isolation of Human Platelets

Human platelets were washed and isolated, as previously described [28,29]. Briefly, fresh whole blood was drawn from healthy donors and anticoagulated with tri-sodium citrate (10.9 mM final concentration) and EGTA (3 mM final concentration). Platelet-rich plasma (PRP) was prepared via centrifugation at 200× *g* for 10 min at room temperature (RT). The supernatant of PRP was diluted with CGS buffer (120 mM NaCl, 12.9 mM trisodium citrate dihydrate, 5 mM D-glucose, pH 6.5) and then centrifuged at 400× *g* for 10 min at RT. The pellet was resuspended in 3 mL CGS buffer and then centrifuged at 400× *g* for 10 min at RT. Finally, the platelet pellet was resuspended in HEPES buffer (150 mM NaCl, 5 mM KCl, 1 mM MgCl_2_, 5 mM D-glucose, 10 mM HEPES, pH 7.4). The platelet concentration was adjusted to 3 × 10^8^/mL, and the platelets were rested for 15 min at 37 °C.

### 4.4. Light Transmission Aggregometry (LTA)

Using an Apact4S Plus aggregometer, washed human platelets (200 µL, 3 × 10^8^/mL) were prepared in cuvettes and treated with 50 ng/mL Cvx or 0.9% NaCl (vehicle control), 0.2 µM PDBu or 0.1% DMSO (vehicle control), or 1 µM OA or 0.1% EtOH (vehicle control) under stirring. Washed human platelets were preincubated with 0.2 µM PDBu or 0.1% DMSO (vehicle control) for 1 min, with 5 µM GFX or 0.1% DMSO (vehicle control) for 5 min, with 5 nM iloprost or 0.9% NaCl (vehicle control) for 5 min, with 1 µM PRT-060318 or 0.1% DMSO for 5 min, or with 10 µM PP2 or 0.1% DMSO for 5 min before 50 ng/mL Cvx stimulation. For Western blotting, aggregation was stopped by adding 100 µL of 3× Lämmli buffer (200 mM Tris/HCl, 15% (*v*/*v*) glycerol, 6% (*w*/*v*) SDS, 0.06% (*w*/*v*) bromophenol blue, 1:10 β-mercaptoethanol) at 0 min, 1 min, 2 min and 5 min. Samples were boiled at 95 °C for 10 min with gentle shaking.

### 4.5. SDS-PAGE and Western Blot Analysis

Samples for Western blotting analysis were prepared by adding 3× Lämmli buffer to washed platelet suspensions. The lysed platelet proteins were separated via electrophoresis using 8% gels, as previously described. Proteins separated via SDS-polyacrylamide electrophoresis were transferred to polyvinylidene difluoride membranes (PDVF). The membranes were blocked with 5% bovine serum albumin (BSA) in 1× TBS-T (20 mM tris, 140 mM NaCl, 0.1% (*v*/*v*) Tween-20) for 1 h at RT, and then incubated overnight with desired antibodies in 1× TBS-T/5% BSA at 4 °C. The incubated membranes were washed three times with 1× TBS-T and treated for 2 h at RT with relevant secondary horseradish peroxidase (HRP)-conjugated antibodies in 1× TBS-T/5% BSA. After three washes with 1× TBS-T, the membranes were developed via electrochemiluminescence (ECL) detection. The phosphoantibodies used were against Btk pS180, pY223, pY551, pSyk pS297, pY352 and pY525/526, PLCγ2 pY759 and pY1217, anti-Syk, anti-Btk and anti-PLCγ2.

### 4.6. Statistical Analysis

Data are represented as the mean ± standard deviation (SD), from n ≥ 3 independent experiments with platelets from at least three healthy donors. Statistical analysis was performed using GraphPad Prism 9.0.1 (GraphPad Software, San Diego, CA, USA). One-way or two-way ANOVA, followed by Sidak’s multiple comparison test, was used for the comparison of two groups. A *p*-value < 0.05 was considered significant.

## Data Availability

All data are included in the manuscript as figures, tables or supplement figures.

## Supplementary Materials

The following supporting information can be downloaded from https://www.mdpi.com/article/10.3390/ijms24097776/s1.
