# Supplementary material for: Differential Regulation of GPVI-Induced Btk and Syk Activation by PKC, PKA and PP2A in Human Platelets
Source: Int J Mol Sci. 2023 Apr 24;24(9):7776. doi: 10.3390/ijms24097776 (PMC10178361; doi:10.3390/ijms24097776)
Supplement: Supplementary file 1 [file ijms-24-07776-s001.zip › ijms-2305921-supplementary.pdf]

## Supplementary Figure

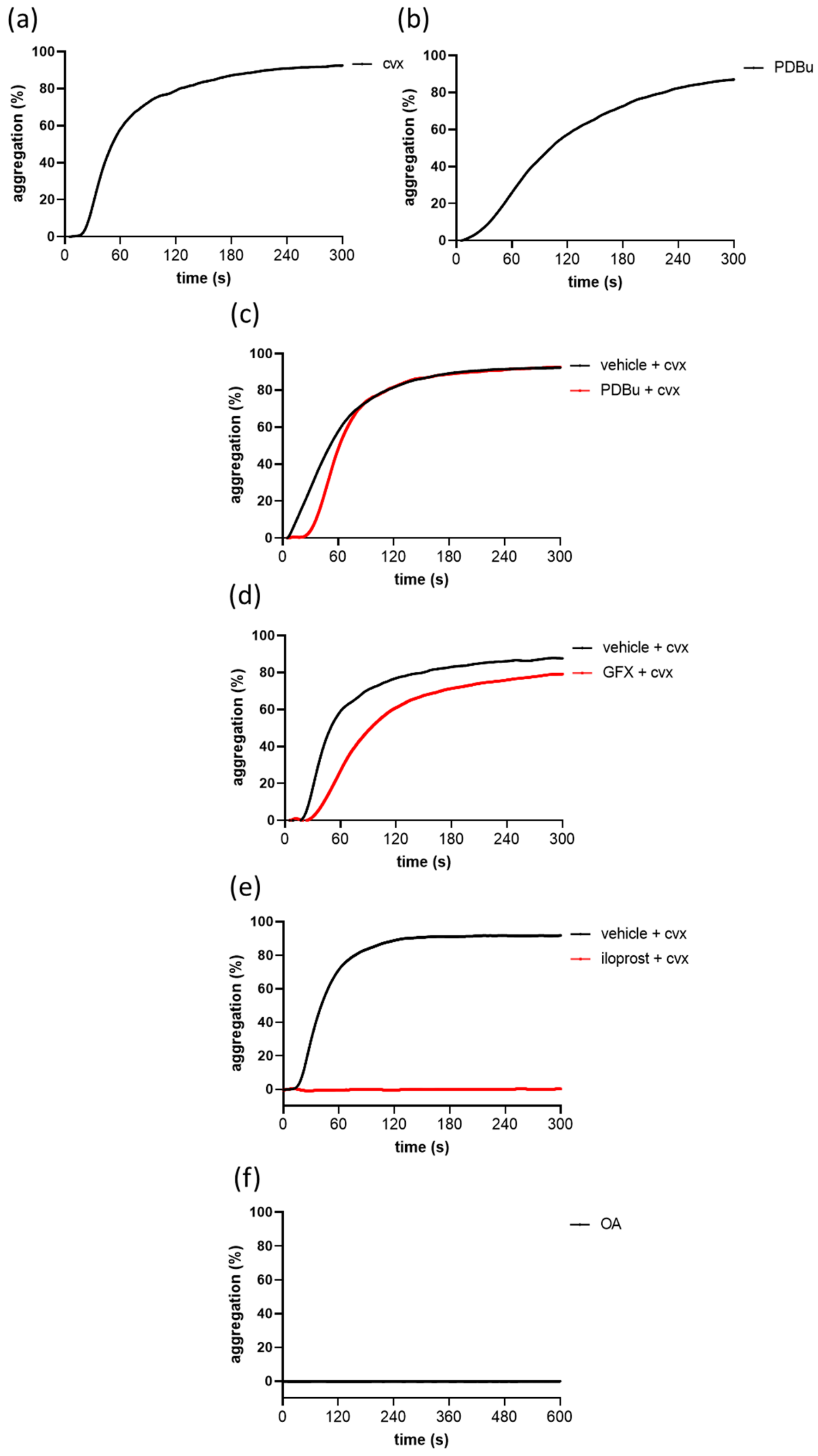


**Figure S1.** Representative aggregation curves of washed human platelets treated with (**A**) convulxin (cvx, 50 ng/ml), (**B**) the PKC activator phorbol 12, 13-dibutyrate (PDBu, 0.2 µM), (**C**) vehicle (0.1% DMSO) plus cvx (50 ng/ml) or 0.2 µM PDBu plus cvx (50 ng/ml), (**D**) vehicle (0.1% DMSO) or 5 µM GF109203X (GFX) for 5 min prior to stimulation with 50 ng/ml cvx, (**E**) vehicle (0.9% NaCl) or iloprost (5 nM) for 5 min prior to stimulation with 50 ng/ml cvx, (**F**) okadaic acid (OA, 1 µM).
